# The tailless gecko gets the worm: prey type alters the effects of caudal autotomy on prey capture and subjugation kinematics

**DOI:** 10.3389/fnbeh.2023.1173065

**Published:** 2023-08-23

**Authors:** Marina F. Vollin, Timothy E. Higham

**Affiliations:** Department of Evolution, Ecology, and Organismal Biology, University of California, Riverside, Riverside, CA, United States

**Keywords:** autotomy, *Coleonyx variegatus*, predator-prey interaction, Gekkota, prey shaking, prey capture

## Abstract

Prey capture and subjugation are complex behaviors affected by many factors including physiological and behavioral traits of both the predator and the prey. The western banded gecko (*Coleonyx variegatus*) is a small generalist predator that consumes both evasive prey items, such as spiders, wasps, and orthopterans, and non-evasive prey items, including larvae, pupae, and isopterans. When consuming certain prey (e.g., scorpions), banded geckos will capture and then rapidly oscillate, or shake, their head and anterior part of their body. Banded geckos also have large, active tails that can account for over 20% of their body weight and can be voluntarily severed through the process of caudal autotomy. However, how autotomy influences prey capture behavior in geckos is poorly understood. Using high-speed 3D videography, we studied the effects of both prey type (mealworms and crickets) and tail autotomy on prey capture and subjugation performance in banded geckos. Performance metrics included maximum velocity and distance of prey capture, as well as velocity and frequency of post-capture shaking. Maximum velocity and distance of prey capture were lower for mealworms than crickets regardless of tail state. However, after autotomy, maximum velocity increased for strikes on mealworms but significantly decreased for crickets. After capture, geckos always shook mealworms, but never crickets. The frequency of shaking mealworms decreased after autotomy and additional qualitative differences were observed. Our results highlight the complex and interactive effects of prey type and caudal autotomy on prey capture biomechanics.

## 1. Introduction

A wide variety of temporary challenges (i.e., perturbations), which can stem from the animal itself (e.g., carrying young) or from the environment, can have significant negative effects on locomotor performance (Jagnandan and Higham, 2018a), potentially resulting in reduced ability to complete ecologically relevant tasks. Although animals might alter their behavior to avoid the negative effects of perturbations, there are many situations where performance is decreased. Autotomy, the voluntary severance of an appendage, is an internal perturbation that can incur a wide variety of costs and even occasionally benefits. Although autotomy is present in a wide variety of invertebrates and amphibians (Emberts et al., 2019), perhaps the best developed, and best studied, example of autotomy is in squamate reptiles. Reptiles, particularly lizards, are capable of complete caudal autotomy and regeneration, constituting up to 25% of total body mass (Gillis and Higham, 2016). Following tail loss, individuals experience negative effects, such as decreased terrestrial locomotion performance (Ballinger et al., 1979; Bateman and Fleming, 2009), lower social standing (Berry, 1974; Fox et al., 1990; Martín and Salvador, 1993), decreased growth and reproduction (Smyth, 1974; Ballinger and Tinkle, 1979; Dial and Fitzpatrick, 1981), and an increased risk of capture when encountering predators in a staged encounter (Congdon et al., 1974; Daniels et al., 1986). Additionally, a mark-recapture study of lizards in the wild found a trend of more complete tails in surviving individuals compared to individuals that died over the course of the study (Wilson, 1992).

However, there are several cases where autotomy either does not have a negative effect or actually results in an increase in performance. For example, *Phyllodactylus marmoratus*, a gecko with a large, fairly immobile tail runs faster after caudal autotomy compared to before [Daniels, 1983, reviewed in McElroy and Bergmann (2013)]. In the social anole species *Anolis sagrei*, dominant males retained their status after tail loss in a captive setting (Kaiser and Mushinsky, 1994), and, in species such as *Uta stansburiana*, tailed individuals were consumed at the same rate as tailless individuals (Althoff and Thompson, 1994; Salvador et al., 1995; Webb, 2006). What allows some individuals to mitigate the negative effects of an internal perturbation while others suffer decreased performance? Addressing this question will provide insight into the evolution of behavioral lability but may also lead to new questions related to the neural adaptations following a traumatic loss of a body part.

How might the neural system be related to tail autotomy? The act of severing a tail constitutes a major biomechanical change for a lizard. Not only does autotomy represent a rapid loss of body mass, but it also shifts the animal’s center of mass forward. Individuals compensate for these changes by altering stride length (Jagnandan and Higham, 2017) and adopting a more sprawled position post-autotomy (Jagnandan et al., 2014; Vollin and Higham, 2021). Additionally, there are significant changes in neuromotor control of hindlimb muscles following autotomy in leopard geckos (Jagnandan and Higham, 2018b). Most propulsive muscles exhibit decreases in activity, suggesting that mass-related sensory signaling can permit short-term shifts in neural control in geckos. Although it is well known that non-mammalian vertebrates, including reptiles, fishes, and amphibians, have extensive abilities to generate new neurons in adulthood (McDonald and Vickaryous, 2018), changes to brain structure following autotomy (both short-term and during regeneration) are less understood. Bradley et al. (2021) found cerebellar structure changed in leopard geckos following autotomy via a change in dendrite diameter and number of dendrite intersections within Purkinje cells. As the cerebellum is a region of the brain involved in motor control, this altering of brain structure may assist in motor adjustments following tail loss (Bradley et al., 2021).

Predation events can generally be divided into five main phases: encounter, detection, pursuit, subjugation and consumption [Endler, 1986; cited in Downes and Shine (2001)], with prey capture referring specifically to events that take place during pursuit and subjugation. Lizards have become a model system for prey capture studies [reviewed in Schwenk (2000), Bels et al. (2019), Montuelle and Kane (2019)], with most studies focusing on how the prey is captured with the jaws or tongue. Measurements within these studies focus on the cranial movements of the skull (Montuelle et al., 2012), but post-cranial movements have received far less attention, despite the clear dependence on locomotor systems in capturing elusive prey (Bels et al., 2019). The few studies that do examine the integration of prey capture and locomotor systems in lizards have only examined the link, or lack thereof, between forelimb and cranial movements (Montuelle et al., 2008). Lizards will often approach prey items closely enough to capture them using only their jaws and forelimbs, but some species, particularly species capturing more agile prey, will lunge at prey from farther away (Moermond, 1981; Montuelle et al., 2008; Bels et al., 2019; Vollin and Higham, 2021). These strikes require running and often include some degree of jumping, two behaviors that are affected by autotomy (Bateman and Fleming, 2009; Gillis et al., 2009; Kuo et al., 2012). For example, the generalist insectivore gecko *Coleonyx variegatus* lunged toward prey, utilizing movements of the hindlimbs to propel the individual an average of 2 cm toward the prey. *C. variegatus* was significantly slower striking at prey after autotomy, although prey capture success rates were unaffected by tail loss (Vollin and Higham, 2021).

In addition to lunging at the prey and securing it with the jaws, prey are often manipulated in order to initiate ingestion. Because dangerous prey can injure a predator following capture, some lizards will utilize a variety of behavior patterns to incapacitate or kill prey, reducing the possibility of escape or injury. Prey shaking is one example and is found in many vertebrates including mammals [reviewed in Eisenberg and Leyhausen (1972)], birds (Schlee, 1986), sharks (Gilbert, 1962), and reptiles (Dauth, 1986; Whitford et al., 2022). Prey shaking is formally defined as “a continuous side-to-side swinging of the head within the functional system [of] “feeding behavior”” (Dauth, 1986). Although oscillations of the head by a predator for the purposes of prey subjugation visually appear to be homologous across many species, the purpose of shaking the prey depends upon the species and context. Its purpose may be to stun or kill the prey (Dauth, 1986), to remove hazardous parts of the prey item, such as stingers or limbs (Whitford et al., 2022), or to break the prey item up into smaller pieces for easier consumption (Gilbert, 1962). Whiptail lizards will modulate their behavior to violently shake scorpions while electing to merely directly consume similarly sized crickets, suggesting these lizards are capable of differentiating between prey types, although the cues used to determine potential danger are unknown (O’Connell and Formanowicz, 1998). Prey shaking behaviors have also been reported in geckos (Whitford et al., 2022), lacertids (Dauth, 1986), and varanids (Loop, 1974).

Western banded geckos (*Coleonyx variegatus*) are a small eublepharid gecko species from the Southwestern United States. This species has large, active tails, and a diverse diet of evasive invertebrates, including scorpions, coleopterans, orthopterans, and isopterans (Parker and Pianka, 1974). In one population of *C. variegatus*, 74% of adults had either missing or regenerated tails, indicating natural rates of tail autotomy are quite high (Parker, 1972). Although *C. variegatus* tails are quick to regenerate, the majority of adults must capture food without a tail at some point in their life, making the question of how tail autotomy affects prey capture ability highly ecologically relevant. Animals will often modulate prey approach and subjugation in response to different prey types, particularly in response to size (Deban, 1997; Schaerlaeken et al., 2007) and mobility (Moermond, 1981; Monroy and Nishikawa, 2011; Montuelle et al., 2012). Capturing agile prey demands faster and often more accurate movements than slower, less mobile prey.

Using banded geckos, we examined capture and post-capture performance using evasive (crickets) and non-evasive (mealworms) prey. We addressed the following questions: 1) Does caudal autotomy differentially alter prey capture performance based on prey type? Based on the typically lower locomotor performance and the unstable lunges observed post-autotomy, we hypothesize that the shift in center of mass after tail loss will result in an increased forward pitch during prey capture lunges, which will result in less accurate and slower strikes. We predict any observed negative impacts of tail autotomy on prey capture kinematics while attacking mobile prey will be significantly reduced or non-existent when attacking the mealworms. 2) Does caudal autotomy influence shaking performance of elongate prey? As prey shaking is a complex and vigorous activity, we predicted that the loss of the tail and accompanying body weight would result in a destabilizing effect on the gecko as it shakes, resulting in either fewer or slower shakes.

## 2. Materials and methods

We tested five juvenile (one female and four male) *C. variegatus* within this study. Outside of trials, geckos were kept in separate tanks at a room temperature between 18.3 and 24.8°C, with either incandescent lights or heat pads for a basking spot. Geckos were fed a mixture of live crickets and mealworms and provided water *ad libitum* but were fasted for 48 h before trials. At the time of testing, the five individuals ranged from 3.23 to 4.03 g in body mass and 48.94–56.91 mm in snout–vent length.

**FIGURE 1:**
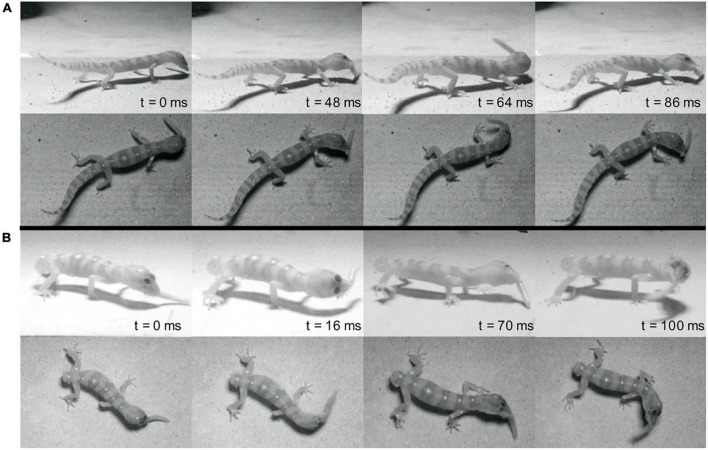
Representative sequences of prey shaking behavior from the same individual before **(A)** and after **(B)** tail autotomy. **(A)** Includes all panels above the center horizontal black line while **(B)** includes all panels below the center horizontal black line. *t* = 0 denotes the onset of shaking. Time is in milliseconds. Note foot position change in panel **(B)**.

We used 3D videography to record banded gecko prey capture strikes on two prey items, crickets (evasive) and mealworms (non-evasive). Prior to placement in the testing arena, landmarks were painted on the gecko’s head, midpoint of the back, and pectoral and pelvic girdles (Figure 1) using white nail polish. Tests were conducted in low light using an IR illuminator to capture the semi-natural behavior of this nocturnal gecko species. Two synchronized high-speed videos shooting at 500 frames per second captured dorsal and lateral views of the focal testing arena. A single gecko was placed into an experimental arena, and trials began by dropping a single prey item into the arena. Approximately 5 min after consumption, another insect was placed in the tank until the gecko no longer pursued the prey item [as in Vollin and Higham (2021)]. A minimum of 24 h after at least three successful and three unsuccessful strikes were recorded for each prey type, gecko tails were fully autotomized by pinching the base of the tail (Jagnandan and Higham, 2018b). The geckos were tested again 2 h after autotomy, 1 day after autotomy, and every other day afterward for 2 weeks. All post-autotomy trials were grouped together in statistical analysis due to small sample size per day. Mealworm and cricket size were not standardized but had little variance and prey size was selected at random for the duration of the trials. All animal procedures were approved by a UCR IACUC protocol (A-20170039). Data from the cricket trials of this study are from Vollin and Higham (2021).

A pre-measured calibration object constructed of LEGO bricks was used to calibrate videos for digitization. Relevant points on the gecko were digitized using the MATLAB DLTdv8 tool (Hedrick, 2008). The resulting coordinates were smoothed using a low-pass zero-lag Butterworth filter at 50 HZ [as in Whitford et al. (2019)] using a custom MATLAB script. Smoothed snout and prey item points were used to calculate the maximum 3D distance between the gecko and prey prior to the initiation of rapid motion toward the prey item. Maximum distance was averaged for each individual before and after autotomy. The snout point was used to calculate maximum velocity of the gecko during the prey strike as well as the maximum velocity and amplitude of the prey shake. These maximum values were averaged for each individual and prey type before and after autotomy. Prey shake frequency was calculated by dividing the total number of prey shake oscillations by the total time shaking the prey item. Shake frequency was averaged for each individual before and after autotomy. Shakes were defined as oscillations of the head from center, to the right and left, and back to center (see Figure 1). However, “half-shakes,” where the head only turned to one side before returning to center, were also included in analyses. In trials where geckos paused during prey shakes, only the first section of shaking was analyzed.

A Wilcoxon Signed-Rank Test was used to compare the rate of prey capture success between pre- and post-autotomy strikes. Analyses of maximum velocity and distance to prey for both cricket and mealworm trials were completed using repeated measures ANOVA tests with prey type and autotomy as fixed effects. Post-hoc analyses were carried out on all pairwise multiple comparison procedures using the Holm-Sidak method. Shake velocity and amplitude analyses were calculated using a generalized linear mixed model with autotomy as fixed effect and individual as a random effect. Calculations were carried out using custom script in RStudio, MATLAB, and JMP. *P* < 0.05 was used as the criterion for statistical significance.

## 3. Results

A total of 140 cricket trials (25–31 trials per individual) and 53 mealworm trials (9–12 trials per individual) were recorded. Geckos were successful in 116 of the 140 cricket trials and in all recorded mealworm trials. Approach and capture of the prey item differed significantly between prey types. When attacking mealworms, geckos approached closely and utilized movements of mainly the head, neck, and forelimbs to maneuver the jaws into position to grasp the prey. In contrast, geckos typically stopped at a greater distance when attacking crickets, pushing off with the hindlimbs to lunge forward and seize the prey. Additionally, geckos only shook the mealworms (see Figure 1), and did so in 51 of 53 trials.

**FIGURE 2:**
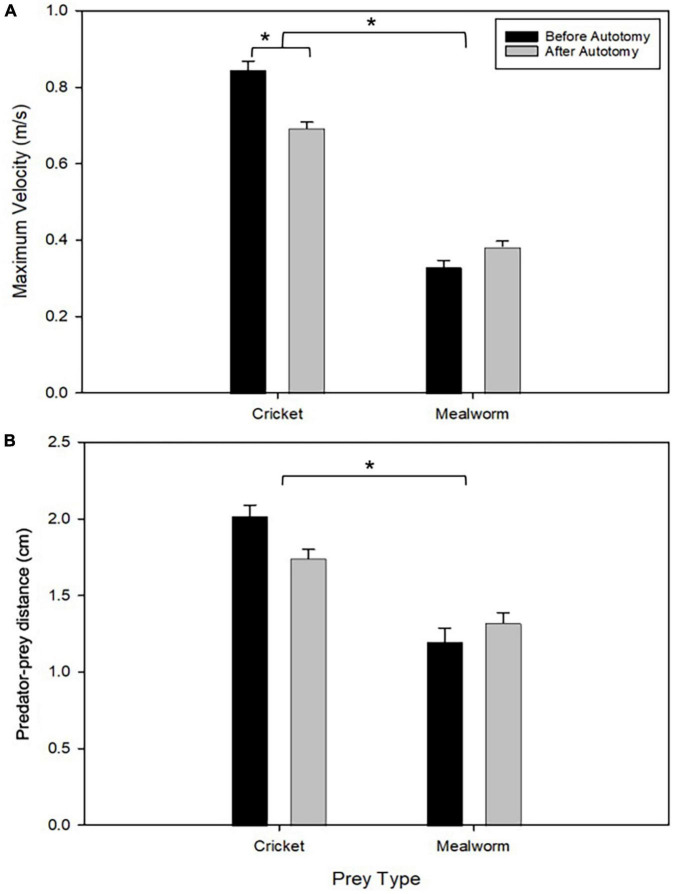
Comparison of maximum velocity **(A)** and starting distance to the prey **(B)** between pre- and post-autotomy trials. Error bars represent standard error of the mean (SEM) and asterisks represent a *P* < 0.05 determined by a two-way repeated measures ANOVA with Holm-Sidak’s multiple comparisons test. (see Table 1).

**FIGURE 3:**
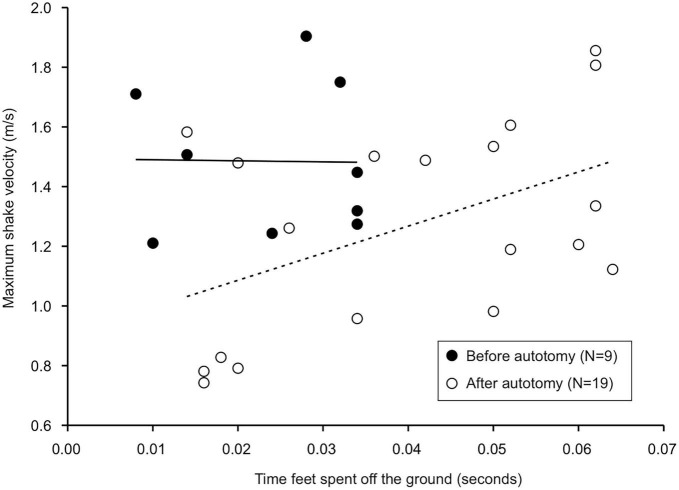
Linear regressions between time gecko feet spent off the ground during the prey shake and maximum prey shake velocity. The time feet spent airborne was positively correlated with maximum shake velocity after, but not before autotomy. Equation of the regression before autotomy was *y* = –0.357x + 1.494, R^2^ = 0.0002, *P* > 0.05. Equation of the regression after autotomy was *y* = 9.08x + 0.090, R^2^ = 0.23, *P* < 0.05.

Tail state did not significantly affect the percentage of successful strikes for cricket (Wilcoxon signed-rank test, *z* = 7, *P* = 1) or mealworm trials (Wilcoxon signed-rank test, *z* = 2, *P* = 0.1875). The maximum velocity of the strike was significantly different across prey type and across tail state. Geckos were slower overall while striking at mealworms (Repeated measures ANOVA, *F* = 154, *p* = 0.0002). However, after autotomy geckos had significantly lower maximum velocities while striking at crickets, (Holm-Sidak, *t* = 4.880, *p* = 0.002) but higher maximum velocities when striking at mealworms (relative to pre-autotomy mealworm trial measures) (Holm-Sidak, *t* = 1.687, *p* = 0.135) (Figure 2A and Table 1). Strike distance was not significantly affected by tail state, but strike distance was significantly shorter in mealworm trials than in cricket trials (Repeated measures ANOVA, *F* = 18.91, *p* = 0.0122) (Figure 2B). Although shake frequency (shakes per second) was significantly lower after tail loss (GLMM, F_1,4_ = 14.183, *P* = 0.020), maximum shake velocity and average amplitude, were not significantly different (GLMM, velocity: F_1,4_ = 2.826, *P* = 0.168, amplitude: F_1,4_ = 0.799, *P* = 0.385) (Table 2). Qualitatively, particularly vigorous prey shakes after autotomy involved the gecko oscillating to the side such that the limbs would often leave the ground, with the gecko often becoming fully airborne (Figure 1B). A linear regression between time the gecko spent with a foot off the ground during the shake and maximum shake velocity was significant after autotomy but not before (Figure 3).

**TABLE 1:** Repeated measures ANOVA values for maximum strike velocity and strike distance.

	Maximum velocity			Strike distance		
Effect	dF	*F*-value	*P*-value	dF	*F*-value	*P-*value
Prey type	1	154	0.000242	1	18.91	0.0122
Autotomy	1	8.037	0.0471	1	0.641	0.468
Prey type × autotomy	1	15.79	0.0165	1	7.123	0.0559

**TABLE 2:** Kinematic data for prey shakes averaged across individuals before and after autotomy.

	Before	After
Maximum shake velocity	1.18 ± 0.17 m/s	1.40 ± 0.19 m/s
Maximum shake amplitude	10.78 ± 1.57 mm	12.35 ± 2.00 mm
Shake frequency	12.30 ± 2.06 shakes/second	19.30 ± 2.66 shakes/second

## 4. Discussion

The effect of tail autotomy on gecko prey capture performance depends on the type of prey being attacked. Although strike velocity was lower in mealworm strikes regardless of tail state, prey capture strikes were slower after tail loss when attacking crickets, but strikes were faster after autotomy when attacking mealworms. The lower velocity in the mealworm trials overall is partially explained by the significantly shorter strike distance. However, strike distance only differed between prey types, not tail state, and does not explain why maximum velocity increased after autotomy in mealworm trials. Our results highlight that the influence of a major internal perturbation, such as tail autotomy, can be context dependent. Foraging strategy and preferred prey type are correlated to brain morphology in lizards (Day et al., 1999). If, as suggested by Bradley et al. (2021), neural alterations play a role in controlling changes in motor output following autotomy, the changes in brain structure following tail loss might depend on feeding ecology and would likely vary across ecologically different species.

### 4.1. The effect of prey type

Mealworms are almost completely stationary and, thus, the risk of losing the prey item is negligible. Therefore, geckos do not need to swiftly lunge and grasp the prey item. As only movements of the head and front limbs are utilized to grasp the mealworm, the tail’s role in this process may be minimal, although the slight increase in strike velocity may be a benefit of the weight loss following autotomy.

Crickets, however, are much more capable of evading capture so the gecko must lunge faster and attack from a greater distance to be successful. When the gecko is engaging its hindlimbs to generate forward propulsive forces, the tail may be an important appendage for counterbalancing the body as it moves through the air (Vollin and Higham, 2021). The mechanism by which tail autotomy may cause a reduction in velocity during these lunge strikes is unknown, but we hypothesize that the change is a result of the more sprawled posture geckos adopt after autotomy (Jagnandan et al., 2014), as well as changes in lower hindlimb muscle recruitment during the lunge to avoid balance issues associated with the shifted center of balance (Vollin and Higham, 2021). Quantifying both motor activity, as well as leg function, when feeding on different prey types will be an important next step.

Moermond (1981) observed a modulation in prey capture movements in five species of *Anolis* lizards similar to the behavior described in this study. Moermond also noted that the “jump-strike” was most often utilized against prey prone to escape and “approach-pause-strikes” (defined as when the individual would closely approach a prey item, pause and orient the head, and rapidly grasp the prey item) were used for non-elusive prey. This modulation has also been observed in other *Anolis* species (Montuelle et al., 2008). Future studies that tease apart the effects of autotomy on prey capture across a range of species that vary in ecology and morphology will determine whether similar performance changes occur when preying on a variety of prey types.

### 4.2. Prey shaking

Prey shaking is a vigorous movement that involves lateral oscillations of the head, forelimbs, and anterior portion of the trunk, with the hindlimbs and tail appearing to serve as anchors during the movement. Although geckos almost always shook the mealworms after capture, both before and after autotomy, the loss of the tail did not have significant effects on most of the kinematic variables measured, with prey shake frequency as the only exception. Variation among individuals was very high for all kinematic variables.

Although this decrease in shake frequency does point to a decrease in performance post-autotomy, the ecological relevance of this decrease is not clear. The purpose of the prey shake in this interaction is likely to stun the prey item into immobility given that the mealworms do not have any defensive structures to be removed and do not break up into smaller pieces for easier consumption during the shake. In previous work, most scorpions were still mobile after being shaken by banded geckos, but the shake may have broken off the stinger or at least limited the amount of venom that could be injected (Whitford et al., 2022). Further research is needed to determine if shaking is effective at damaging the prey item. If the purpose of the shake is to slam the mealworm against the substrate hard enough to incapacitate it, maximum shake velocity would be a more important measurement of performance compared to shake frequency.

Although few kinematic variables of the prey shake were significantly different after autotomy, we observed several qualitative differences between shakes after tail loss. Post-autotomy many of the most vigorous shakes were accompanied by increased rotation of the trunk and posterior end of the body, resulting in the hindlimbs leaving the ground for a portion of the shake. We hypothesize the tail may be acting as a counterbalance for the body during the oscillations and that the loss of the tail and associated shift in center of mass may have a destabilizing effect on the gecko when it attempts to perform a prey shake. With the mealworms, this instability was visible in the limbs coming off the ground, but the geckos may have compensated for this instability by reducing average velocity of the shake. We found a positive correlation between the time that the back legs spent off the ground and maximum velocity of the shake after autotomy, but not before, indicating balance may be more coupled to shake velocity after tail loss (Figure 3).

**FIGURE 4:**
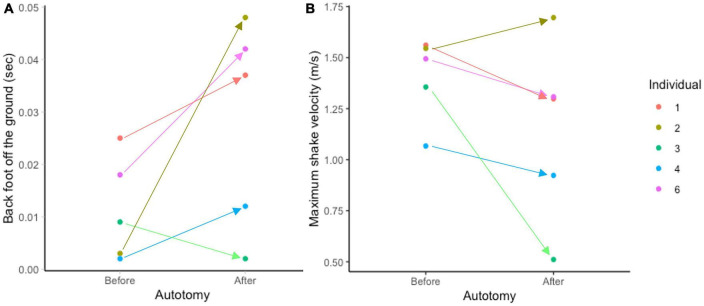
Average time at least one back foot spends airborne **(A)** and maximum velocity of the prey shake **(B)** by individual in mealworm trials. Arrows indicate difference between pre- and post-autotomy trials. Note individual 2 has the largest increase in time in panel **(A)** and is the only individual to have increased performance in panel **(B)** while individual 3 is the only individual to spend less time with its foot off the ground after autotomy in panel **(A)** and experiences the largest decrease in performance in panel **(B)**.

This relationship points to a tradeoff geckos face post-autotomy: to perform a faster, more effective shake but become unbalanced during the oscillations, or reduce velocity to perform a less vigorous shake. The variation observed among individuals supports the existence of this trade-off. The individual that experienced the sharpest increase in time the limbs spent off the ground was also the only individual to have a higher maximum shake velocity post-autotomy, while the only individual to spend less time with its limbs off the ground post-autotomy also experienced the sharpest drop in maximum shake velocity after tail loss (Figure 4). This tradeoff is not likely to have an impact on geckos in nature since mealworms can neither escape nor harm the gecko and do not need to be broken down to be efficiently consumed. However, western banded geckos also prey on dangerous prey such as the dune scorpion (*Smeringurus mesaensis*) (Whitford et al., 2022). Thus, the shaking behavior that we observed may simply reflect the gecko responding to the potential danger of a different elongated prey, such as a scorpion. Previous data suggest geckos may be shaking scorpions nearly twice as fast as the maximum velocities recorded in our study (Whitford et al., 2022). Thus, future studies should examine how tail autotomy impacts these faster prey shakes on a dangerous prey item. We predict that, in predation events where shaking the prey is essential to safely and effectively consuming the prey, autotomy will have a significant negative effect on the gecko’s ability to successfully capture and consume the prey because of the tradeoff between shake velocity and shake stability that is evident post- autotomy.

## 5. Conclusion

Our results underscore the complexity of prey capture behaviors, and the effects that internal perturbations can have on maximum strike velocity. We observed that tail autotomy negatively impacted performance of banded geckos while attacking crickets but slightly improved performance when consuming mealworms. This differing effect of autotomy on the capture of different prey items points to a potential strategy geckos may employ in the wild after they have lost their tail. As banded geckos regularly capture prey types that range widely in mobility, these geckos may be able to offset the negative impacts of tail loss by temporarily favoring less mobile prey items. The mechanism by which autotomy reduces the maximum velocity of prey capture strikes when attacking elusive prey remains unknown and future directions should endeavor to determine the biomechanical basis for the changes occurring after autotomy. Going a step deeper, as the biomechanical adjustments made to terrestrial locomotion after autotomy may be driven by a neurological change (Bradley et al., 2021), it is likely these changes to prey capture lunges are also accompanied by a change in brain structure. Elucidating the neurological mechanism behind this drop in performance would provide valuable insight into the links between neurological control, movement, and temporary internal perturbations.

## Data availability statement

The raw data supporting the conclusions of this article will be made available by the authors, without undue reservation.

## Ethics statement

The animal study was approved by the UC Riverside Institutional Animal Care and Use Committee. The study was conducted in accordance with the local legislation and institutional requirements.

## Author contributions

MV collected and analyzed the data and wrote the initial version of the manuscript. Both authors designed the experiments, revised and contributed to the article and approved the submitted version.

## References

[B1] Althoff D. M. Thompson J. N. (1994). The effects of tail autotomy on survivorship and body growth of *Uta stansburiana* under conditions of high mortality. *Oecologia* 100 250–255. 10.1007/BF00316952 28307008

[B2] Ballinger R. E. Tinkle D. W. (1979). On the cost of tail regeneration to body growth in lizards. *J. Herpetol.* 13 374–375. 10.1007/s00442-021-05084-6 34907460 PMC9056467

[B3] Ballinger R. E. Nietfeldt J. W. Krupa J. J. (1979). An experimental analysis of the role of the tail in attaining high running speed in *Cnemidophorus sexlineatus* (Reptilia: Squamata: Lacertilia). *Herpetologica* 35 114–116.

[B4] Bateman P. W. Fleming P. A. (2009). To cut a long tail short: A review of lizard caudal autotomy studies carried out over the last 20 years. *J. Zool.* 277 1–14. 10.1111/j.1469-7998.2008.00484.x

[B5] Bels V. L. Pallandre J.-P. Charlier S. Legreneur P. Russell A. P. Paindavoine A.-S. (2019). “Predatory behavior in lizards,” in *Behavior of lizards: Evolutionary and mechanistic perspectives*, eds Bels V. L. Russell A. P. (Boca Raton, FL: CRC Press), 87–105.

[B6] Berry K. H. (1974). The ecology and social behavior of the chuckwalla, *Sauromalus obesus obesus* Baird. *Univ. Calif. Publ. Zool*. 101 1–60.

[B7] Bradley S. S. Howe E. Bailey C. D. C. Vickaryous M. K. (2021). The dendrite arbor of purkinje cells is altered following to tail regeneration in the leopard gecko. *Integr. Comp. Biol.* 61 370–384. 10.1093/icb/icab098 34038505

[B8] Congdon J. D. Vitt L. J. King W. W. (1974). Geckos: Adaptive significance and energetics of tail autotomy. *Science* 184 1379–1380. 10.1126/science.184.4144.1379 4833262

[B9] Daniels C. B. (1983). Running: An escape strategy enhanced by autotomy. *Herpetologica* 39 162–165.

[B10] Daniels C. B. Flaherty S. P. Simbotwe M. P. (1986). Tail size and effectiveness of autotomy in a lizard. *J. Herpetol.* 20 93–96.

[B11] Dauth J. (1986). “On preyshaking (“death-shaking”) in Lacertidae,” in *Studies in herpetology*, ed. Rocek Z. (Prague: Charles University), 593–596.

[B12] Day L. B. Crews D. Wilczynski W. (1999). Relative medial and dorsal cortex volume in relation to foraging ecology in congeneric lizards. *Brain Behav. Evol.* 54 314–322. 10.1159/000006631 10681602

[B13] Deban S. (1997). Modulation of prey-capture behavior in the plethodontid salamander *Ensatina eschscholtzii*. *J. Exp. Biol.* 200 1951–1964. 10.1242/jeb.200.14.1951 9319864

[B14] Dial B. E. Fitzpatrick L. C. (1981). The energetic costs of tail autotomy to reproduction in the lizard *Coleonyx brevis* (Sauria: Gekkonidae). *Oecologia* 51 310–317. 10.1007/BF00540899 28310013

[B15] Downes S. Shine R. (2001). Why does tail loss increase a lizard’s later vulnerability to snake predators? *Ecology* 82 1293–1303. 10.2307/2679990

[B16] Eisenberg J. F. Leyhausen P. (1972). The phylogenesis of predatory behavior in mammals. *Z. Tierpsychol.* 30 59–93. 10.1111/j.1439-0310.1972.tb00844.x 5063891

[B17] Emberts Z. Escalante I. Bateman P. W. (2019). The ecology and evolution of autotomy: Ecology and evolution of autotomy. *Biol. Rev.* 94 1881–1896. 10.1111/brv.12539 31240822

[B18] Endler J. (1986). “Defense against predators,” in *Predator–prey relationships*, eds Feder M. E. Lauder G. V. (Chicago, IL: University of Chicago Press), 109–134.

[B19] Fox S. F. Heger N. A. Delay L. S. (1990). Social cost of tail loss in *Uta stansburiana*: Lizard tails as status-signalling badges. *An. Behav.* 39 549–554. 10.1016/S0003-3472(05)80421-X

[B20] Gilbert P. W. (1962). The behavior of sharks. *Sci. Am.* 207 60–68. 13898543 10.1038/scientificamerican0762-60

[B21] Gillis G. B. Higham T. E. (2016). Consequences of lost endings: Caudal autotomy as a lens for focusing attention on tail function during locomotion. *J. Exp. Biol.* 219 2416–2422. 10.1242/jeb.124024 27535984

[B22] Gillis G. B. Bonvini L. A. Irschick D. J. (2009). Losing stability: Tail loss and jumping in the arboreal lizard *Anolis carolinensis*. *J. Exp. Biol.* 212 604–609. 10.1242/jeb.024349 19218510

[B23] Hedrick T. L. (2008). Software techniques for two- and three-dimensional kinematic measurements of biological and biomimetic systems. *Bioinspir. Biomim*. 3:034001. 10.1088/1748-3182/3/3/034001 18591738

[B24] Jagnandan K. Higham T. E. (2017). Lateral movements of a massive tail influence gecko locomotion: An integrative study comparing tail restriction and autotomy. *Sci. Rep.* 7:10865. 10.1038/s41598-017-11484-7 28883491 PMC5589804

[B25] Jagnandan K. Higham T. E. (2018a). How rapid changes in body mass affect the locomotion of terrestrial vertebrates: Ecology, evolution and biomechanics of a natural perturbation. *Biol. J. Linn. Soc.* 124 279–293. 10.1093/biolinnean/bly056

[B26] Jagnandan K. Higham T. E. (2018b). Neuromuscular control of locomotion is altered by tail autotomy in geckos. *J. Exp. Biol.* 221:jeb179564. 10.1242/jeb.179564 30026242

[B27] Jagnandan K. Russell A. P. Higham T. E. (2014). Tail autotomy and subsequent regeneration alter the mechanics of locomotion in lizards. *J. Exp. Biol.* 217 3891–3897. 10.1242/jeb.110916 25267844

[B28] Kaiser B. W. Mushinsky H. R. (1994). Tail loss and dominance in captive adult male *Anolis sagrei*. *J. Herpetol.* 28 342–346. 10.2307/1564533

[B29] Kuo C.-Y. Gillis G. B. Irschick D. J. (2012). Take this broken tail and learn to jump: The ability to recover from reduced in-air stability in tailless green anole lizards (*Anolis carolinensis)* (Squamata: Dactyloidae)]. *Biol. J. Linn. Soc.* 107 583–592. 10.1111/j.1095-8312.2012.01958.x

[B30] Loop M. S. (1974). The effect of relative prey size on the ingestion behavior of the Bengal monitor, *Varanus bengalensis* (Sauria: Varanidae). *Herpetologica* 30 123–127.

[B31] Martín J. Salvador A. (1993). Tail loss and foraging tactics of the Iberian rock lizard, *Lacerta monticola*. *Oikos* 66 318–324. 10.2307/3544820

[B32] McDonald R. P. Vickaryous M. K. (2018). Evidence for neurogenesis in the medial cortex of the leopard gecko, *Eublepharis macularius*. *Sci. Rep.* 8:9648. 10.1038/s41598-018-27880-6 29941970 PMC6018638

[B33] McElroy E. J. Bergmann P. J. (2013). Tail autotomy, tail size, and locomotor performance in lizards. *Physiol. Biochem. Zool.* 86 669–679. 10.1086/673890 24241064

[B34] Moermond T. C. (1981). Prey-attack behavior of *Anolis* lizards. *Z. Tierpsychol.* 56 128–136. 10.1111/j.1439-0310.1981.tb01291.x

[B35] Monroy J. A. Nishikawa K. (2011). Prey capture in frogs: Alternative strategies, biomechanical trade-offs, and hierarchical decision making. *J. Exp. Zool.* 315 61–71. 10.1002/jez.601 20309849

[B36] Montuelle S. J. Kane E. A. (2019). “Food capture in vertebrates: A complex integrative performance of the cranial and postcranial systems,” in *Feeding in vertebrates*, eds Bels V. L. Whishaw I. Q. (Switzerland: Springer Nature), 71–137. 10.1007/978-3-030-13739-7

[B37] Montuelle S. J. Daghfous G. Bels V. L. (2008). Effect of locomotor approach on feeding kinematics in the green anole (*Anolis carolinensis*). *J. Exp. Zool.* 309A 563–567. 10.1002/jez.484 18661471

[B38] Montuelle S. J. Herrel A. Libourel P.-A. Daillie S. Bels V. L. (2012). Flexibility in locomotor- feeding integration during prey capture in varanid lizards: Effects of prey size and velocity. *J. Exp. Biol.* 215 3823–3835. 10.1242/jeb.072074 22899521

[B39] O’Connell D. J. Formanowicz D. R. (1998). Differential handling of dangerous and non-dangerous prey by naïve and experienced Texas spotted whiptail lizards, *Cnemidophorus gularis*. *J. Herpetol.* 32 75–79. 10.2307/1565482

[B40] Parker W. S. (1972). Aspects of the ecology of a Sonoran Desert population of the western banded gecko, *Coleonyx variegatus* (Sauria, Eublepharinae). *Am. Midl. Nat.* 88 209–224. 10.2307/2424499

[B41] Parker W. S. Pianka E. R. (1974). Further ecological observations on the western banded gecko, *Coleonyx variegatus*. *Copeia* 1974 528–531. 10.2307/1442544

[B42] Salvador A. Martin J. López P. (1995). Tail loss reduces home range size and access to females in male lizards, *Psammodromus algirus*. *Behav. Ecol.* 6 382–387. 10.1093/beheco/6.4.382

[B43] Schaerlaeken V. Meyers J. J. Herrel A. (2007). Modulation of prey capture kinematics and the role of lingual sensory feedback in the lizard *Pogona vitticeps*. *Zoology* 110 127–138. 10.1016/j.zool.2006.09.002 17368008

[B44] Schlee M. A. (1986). Avian predation on heteroptera: Experiments on the European blackbird *Turdus m. merula* L. *Ethology* 73 1–18. 10.1111/j.1439-0310.1986.tb00995.x

[B45] Schwenk K. (2000). “Feeding in lepidosaurs,” in *Feeding: Form, function and evolution in tetrapod vertebrates*, ed. Schwenk K. (San Diego CA: Academic Press), 175–291.

[B46] Smyth M. (1974). Changes in the fat scores of the skinks *Morethia boulengeri* and *Hemiergis peronii* (Lacertilia). *Aust. J. Zool.* 22 135–145. 10.1071/ZO9740135

[B47] Vollin M. F. Higham T. E. (2021). Tail autotomy alters prey capture performance and kinematics, but not success, in banded geckos. *Integr. Comp. Biol.* 61 538–549. 10.1093/icb/icab076 33988701

[B48] Webb J. K. (2006). Effects of tail autotomy on survival, growth and territory occupation in free ranging juvenile geckos (*Oedura lesueurii*). *Austral. Ecol.* 31 432–440. 10.1111/j.1442-9993.2006.01631.x

[B49] Whitford M. D. Freymiller G. A. Higham T. E. Clark R. W. (2019). Determinants of predation success: How to survive an attack from a rattlesnake. *Funct. Ecol.* 33 1099–1109. 10.1111/1365-2435.13318

[B50] Whitford M. D. Freymiller G. A. Higham T. E. Clark R. W. (2022). Shaking things up: The unique feeding behaviour of western banded geckos when consuming scorpions. *Biol. J. Linn. Soc.* 135 533–540. 10.1093/biolinnean/blab167

[B51] Wilson B. S. (1992). Tail injuries increase the risk of mortality in free-living lizards (*Uta stansburiana*). *Oecologia* 92 145–152. 10.1007/BF00317275 28311825

